# Tetrameric Structure of Centromeric Nucleosomes in Interphase *Drosophila* Cells

**DOI:** 10.1371/journal.pbio.0050218

**Published:** 2007-07-31

**Authors:** Yamini Dalal, Hongda Wang, Stuart Lindsay, Steven Henikoff

**Affiliations:** 1 Fred Hutchinson Cancer Research Center, Seattle, Washington, United States of America; 2 Howard Hughes Medical Institute, Seattle, Washington, United States of America; 3 Biodesign Institute, Arizona State University, Tempe, Arizona, United States of America; University of California San Diego, United States of America

## Abstract

Centromeres, the specialized chromatin structures that are responsible for equal segregation of chromosomes at mitosis, are epigenetically maintained by a centromere-specific histone H3 variant (CenH3). However, the mechanistic basis for centromere maintenance is unknown. We investigated biochemical properties of CenH3 nucleosomes from Drosophila melanogaster cells. Cross-linking of CenH3 nucleosomes identifies heterotypic tetramers containing one copy of CenH3, H2A, H2B, and H4 each. Interphase CenH3 particles display a stable association of approximately 120 DNA base pairs. Purified centromeric nucleosomal arrays have typical “beads-on-a-string” appearance by electron microscopy but appear to resist condensation under physiological conditions. Atomic force microscopy reveals that native CenH3-containing nucleosomes are only half as high as canonical octameric nucleosomes are, confirming that the tetrameric structure detected by cross-linking comprises the entire interphase nucleosome particle. This demonstration of stable half-nucleosomes in vivo provides a possible basis for the instability of centromeric nucleosomes that are deposited in euchromatic regions, which might help maintain centromere identity.

## Author Summary

The octameric structure of eukaryotic nucleosomes is universally accepted as the basic unit of chromatin. This is certainly the case for the vast bulk of nucleosomes; however, there have been no reports of the in vivo structure of nucleosomes associated with centromeres. Though centromeres make up only a minute fraction of the genomic landscape, their role in segregating chromosomes during mitosis is essential for maintaining genomic integrity. We report the characterization of centromeric chromatin from *Drosophila* cells, using detailed biochemical, electron microscopic, and atomic force microscopic analyses. Surprisingly, we found that, in striking contrast to bulk chromatin, centromeric nucleosomes are stable heterotypic tetramers in vivo, with one copy of CenH3 (the centromere-specific H3 variant), H2A, H2B, and H4 each, wrapping one full turn of DNA at interphase (the cell growth phase of the cell cycle). This results in nucleosome particles that are only half as high as bulk nucleosomes. These unexpected findings can help account for the dynamic behavior of CenH3-containing nucleosomes, whereby they are deposited promiscuously but are turned over in noncentromeric regions. Our demonstration of the existence of stable half-nucleosomes at centromeres suggests a novel mechanism for maintaining centromere identity.

## Abstract 3

The centromeric nucleosomes of *Drosophila* are histone tetramers rather than the canonical octomer of the rest of chromatin. This unprecedented arrangement of stable half-nucleosomes might help maintain centromere identity.

## Introduction

Centromeres are specialized chromatin structures within eukaryotic chromosomes that ensure equal segregation of daughter chromosomes during mitosis [1]. Centromere function requires a centromere-specific histone H3 (CenH3) variant that replaces H3 in centromeric nucleosomes [2]. CenH3 is thought to determine centromeric identity, because other kinetochore proteins depend on its presence to recognize and localize to the centromere. Consistent with the idea that CenH3 uniquely specifies the active centromere's location, human CenH3 (CENP-A) is enriched at spontaneously occurring active neocentromeres and depleted from the inactive old centromere [1]. Furthermore, the overexpression of CENP-A is correlated with neocentromeres in some human cancers [3], and overexpression of *Drosophila* CenH3 (Cid) leads to its deposition in active euchromatin [4,5,6] and the formation of ectopic centromeres [5]. The ready formation of neocentromeres, which would lead to immediate chromosome loss, is evidently prevented by the active removal of CenH3 deposited in chromosome arms and its degradation in the proteosome [6,7]. It thus appears that centromere identity is maintained, at least in part, by the removal of CenH3 nucleosomes that are deposited in chromosome arms while retaining newly deposited CenH3 nucleosomes at centromeres, which are embedded in a quiescent heterochromatic environment [8].

CenH3s are assembled into nucleosomes independent of replication [9,10,11,12]. At the structural level, CenH3s take the place of histone H3 within nucleosomes, co–immunoprecipitate with core histones H2A, H2B, and H4 from chromatin, and assemble into nucleosome-like particles in vitro [13,14,15,16,17]. However, nuclease digestion of yeast centromeric chromatin yields noncanonical “smeary” digestion patterns, with unusual core protection of 250 bp in budding yeast or reduced protection of approximately 120 bp of DNA in fission yeast [18,19,20], suggesting that centromeric chromatin has unusual nucleosomal properties.

To elucidate features of CenH3 nucleosomes that might help distinguish centromeric chromatin from the rest of the chromosome in vivo, we performed detailed biochemical and structural analyses of native CenH3 chromatin from D. melanogaster cells in culture. Our results indicate that at interphase, CenH3 nucleosomes are stable tetramers, with one copy of CenH3, H2A, H2B, and H4 each. CenH3 nucleosomes wrap one full turn of DNA, form chromatin that appears resistant to condensation, and are visualized as native particles that are only half as high as bulk nucleosomes. This example of a stable half-nucleosome at interphase suggests an inherently incomplete structure that facilitates the removal of CenH3-containing nucleosomes from active euchromatic regions to help maintain centromeres at a single location on the chromosome.

## Results

### A CenH3-Containing Tetramer Is Cross-Linked In Vivo

We first investigated the organization of CenH3 and core histones within centromeric (CenH3) and bulk (BC) chromatin in chromatin extracts from nuclei of *Drosophila* S2 cells, using the primary amine cross-linking agent dimethyl suberimidate (DMS) to stabilize nucleosomal interactions. This compound has been used widely to elucidate histone contacts within histone H1-stripped sucrose-gradient purified chromatin fibers, and in DNA-free histone complexes [21,22].

**Figure 1:**
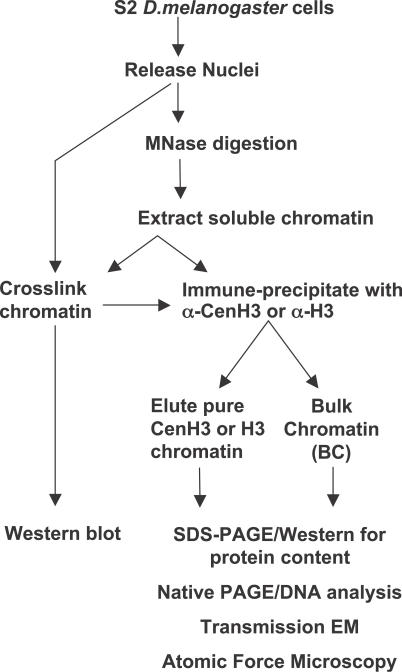
Scheme to Analyze CenH3 Chromatin D. melanogaster S2 or Kc cells were lysed gently with NP-40 to release nuclei. Nuclei were digested over a time course with MNase to release soluble chromatin extracted in 0.35 M salt buffer. Chromatin extracts and intact nuclei were used for protein cross-linking with 1 mg/ml DMS over a time course of 0–3 h. Western blot analysis of H3- and CenH3- nucleosomal complexes was performed on complexes resolved on gradient gels. Soluble chromatin extracts were used for immune-purification of native and in situ cross-linked CenH3-nucleosomes. Immunoprecipitations were subsequently analyzed by 18% SDS-PAGE, native PAGE, MNase digestion, and electron and atomic force microscopy.

**Figure 2:**
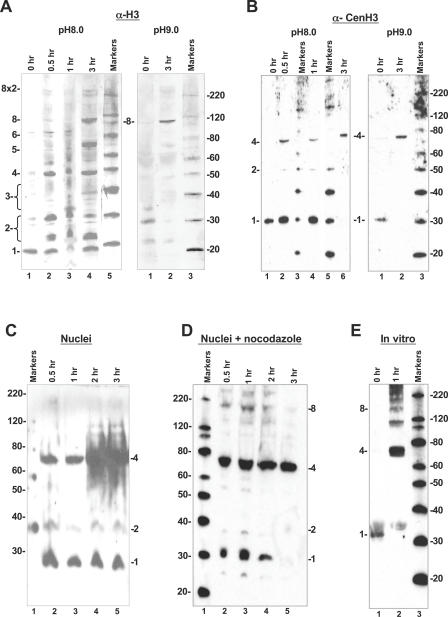
Intranucleosomal Contacts Are Limited in CenH3 Nucleosomes Western blots of D. melanogaster S2 chromatin extracts (A and B), S2 nuclei (C and D), and in vitro reconstituted CenH3 particles (E). Cross-linking was performed with 1 mg/ml DMS at pH 8.0 (left panels in A and B and panels C–E) or pH 9.0 (right panels in A and B) for the indicated times. Samples were resolved on 4%–12% NuPAGE or Tris-Glycine gradient gels. (A) Anti-H3 antibody detects enrichment of the limit octameric species over the cross-linking range in bulk chromatin extracts at pH 8.0 (left panel), and demonstrates enrichment of the octameric species at 110 kDa at the most cross-linked time point in lane 2 at pH 9.0 (right panel). Species annotated 1–8 refer to cross-linked monomer, dimer, and so on up to octamer. (B) Anti-CenH3 antibody probing duplicated samples (as in A) detects only two cross-linked products, deduced to be dimers and heterotypic tetramers (left panel, pH 8.0). Cross-linking to 3 h in pH 9.0 buffer demonstrates a conversion of most of the CenH3 to the species migrating at about 68 kDa. (C) Anti-CenH3 antibody detects robust amounts of a tetrameric species at approximately 68 kDa in cross-linked nuclei (lanes 2–5) obtained from asynchronous cells in culture. (D) Anti-CenH3 antibody detects predominantly tetrameric species at 68 kDa, as well as minor amounts of higher species (lanes 2–5) in cross-linked nuclei obtained from cells arrested at mitosis with nocodazole. (E) Anti-CenH3 antibody detects a cross-linking ladder of in vitro assembled salt-extracted native histones yielding robust amounts of species that are larger than tetramers (lane 2) in 1 h of cross-linking at pH 8.0. A similar range of products was obtained for in vitro assembled particles using recombinant CenH3 (Figure S1B).

DMS was added directly to crude chromatin extracts solubilized by micrococcal nuclease (MNase) digestion of nuclei (Figure 1) under conditions similar to those used in prior studies [21,22] (0.1mg DMS per ml chromatin, pH 8.0 [Figure 2A and 2B, left] or pH 9.0 [Figure 2A and 2B, right]). Under these conditions, cross-links between individual histones within nucleosomes are favored, with cross-linkable lysines being most reactive at pH 9.0, thus enriching the terminal octameric unit of chromatin structure in vivo [21]. Consequently, in our analysis of bulk nucleosomes in chromatin extracts using an anti–N-terminal H3 antibody (Figure 2A: left, lanes 1–4; right, lanes 1–2), the limit octameric (8) species migrating at ∼110 kDa is apparent within 1 h of cross-linking and strongly enriched at 3 h, indicating robust cross-linking of the H3 nucleosome in vivo. The predicted molecular weight of the limit octameric species (110 kDa) is compatible with the apparent molecular weight deduced from the molecular weight markers on the blot (Table 1). Over a shorter time span, we also observed substantial amounts of intermediate particles, such as dimers, trimers, co-migrating homotypic and heterotypic tetramers, and hexamers (Figure S1A) (2 = H3:H4, 3 = H3:H4:H2B, 4 = H2A:H2B:H3:H4, 4* = H3_2_:H4_2_, 6 = 4*:H2A:H2B, 8 = H2A_2_:H2B_2_:H3_2_:H4_2_, respectively, summarized in Table 1). Therefore, in our extracts, the predominant cross-linked form is the octameric species (i.e., 2:2:2:2) [22].

**Table 1:**
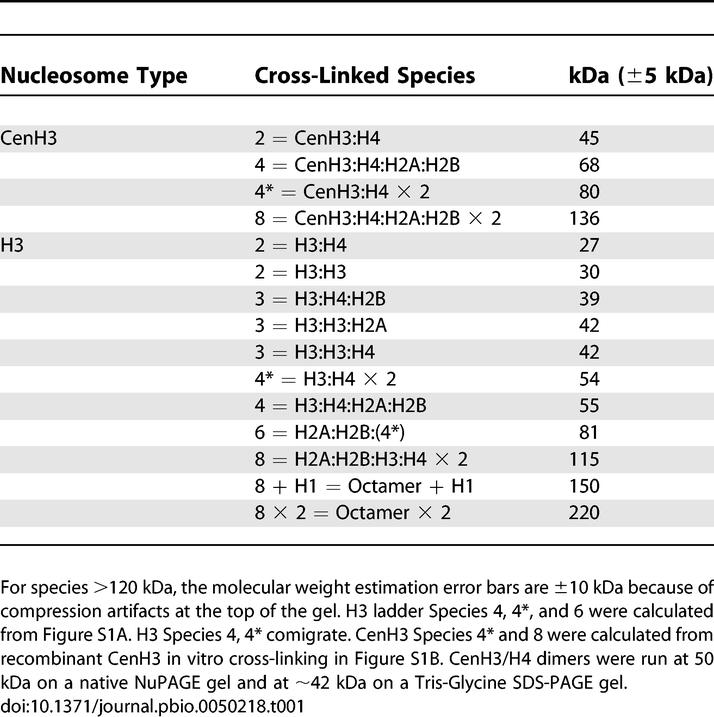
Apparent Molecular Weights of Cross-Linked Histone Complexes

Previous work has shown that CenH3 associates with H2A, H2B, and H4, but not H3 in *Drosophila* chromatin [23] and in budding yeast [24]. We directly assessed histone contacts within CenH3-nucleosomes on the same samples of cross-linked chromatin extracts (as in Figure 2A), and we electrophoresed those samples longer to better resolve the upper part of the gel. For protein blot detection of CenH3 in chromatin extracts and nuclei, we used a fly-specific anti–N-terminal Cid antibody [25]. Surprisingly, across the same cross-linking range used for bulk chromatin, CenH3 (Figure 2B) shows only two major intranucleosomal species, at ∼48 kDa and ∼68 kDa (Figure 2B: left, lanes 1–6), and almost all the CenH3 is converted to the 68-kDa band at 3 h (Figure 2B: right, lanes 1–2). Cross-linking across a shorter time span also detects only ∼48-kDa and ∼68-kDa bands as the dominant cross-linked CenH3 species (Figure S1).

Based on electrophoretic migration relative to markers, the identity of the cross-linked CenH3 complexes can be presumptively assigned (Figure 2B and Table 1): CenH3 (28.5 kDa) cross-links with H4 (11.5 kDa) to yield the 42- to 48-kDa dimer species, then cross-links with H2A/H2B (i.e., +28 kDa) to convert into a heterotypic “4” tetrameric species at approximately 68 kDa. It is unlikely that the 68-kDa band is a homotypic tetramer (CenH3_2_:H4_2_), because the presence of two CenH3s in a homotypic tetramer should yield an approximately 80-kDa, rather than a 68-kDa, product. However, anomalous migration of histone complexes might cause the homotypic form to migrate faster relative to nonhistone protein standards. In either case, the enrichment of a tetramer species as the limit cross-linked product, in contrast to the canonical octameric species in the corresponding bulk chromatin, indicates that the CenH3 nucleosome is highly altered in vivo.

To exclude the possibility that chromatin extraction or dilution impairs the detection of a canonical CenH3 cross-linking ladder, we also surveyed CenH3 contacts directly within nuclei. The concentration of chromatin is >10-fold higher in situ than in extracts (such as in Figure 2A and 2B), therefore chromatin nonhistone protein interactions and internucleosomal interactions are likely to be enriched in situ relative to interactions in extracts. Nuclei were soaked in buffer supplemented with DMS, and cross-linking was allowed to proceed for 1 and 3 h and samples processed as before. Probing with antibody to H3 in bulk chromatin in situ under these conditions detected several non-nucleosomal cross-linked interactions (unpublished data), making assessment of canonical nucleosomal species difficult. Nevertheless, probing in situ cross-linked samples with antibody to CenH3 still yielded only two cross-linked species at 42 kDa and 68 kDa, (assigned “2” and “4”). Within 3 h, as in chromatin extracts, the 68-kDa species was vastly enriched (Figure 2C, lanes 2–5), suggesting it is the primary stable nucleosomal product in vivo.

We also performed the same experiment in cells arrested with nocodazole to enrich for highly compacted chromosomes in mitotic cells (Figure 2D). Under these conditions, in addition to the dominant 68-kDa species, we also observed enrichment of a higher–molecular weight species (Figure 2D, lanes 3 and 4) at approximately 140 kDa. This species is consistent with an octameric product or might reflect a kinetochore protein-associated CenH3 complex. These data suggest that CenH3 cross-linking kinetics in chromatin extracts and in situ are similar, yielding predominantly a CenH3 tetrameric nucleosomal species but no canonical octameric species at interphase.

### Reconstituted CenH3:H4:H2A:H2B Particles Display Multiple Histone Contacts In Vitro

One possible explanation for the apparent absence of higher cross-linked CenH3 species is the relative inability of DMS to cross-link CenH3 homotypic tetramers or octameric species (*Drosophila* CenH3 has three cross-linkable lysines within the core, compared with five lysines within the H3 core). Another possibility is that the antibody fails to recognize its epitope because of epitope masking. To exclude these potential artifacts, we incubated endogenous histones obtained from 2 M NaCl histone extracts from Kc cells (Figure 2E) or from recombinant CenH3, H2A, H2B, and H4 histones (Figure S1B) in equimolar amounts, and raised the salt concentration from low to high salt (salt step). Under these conditions, in the absence of DNA, histones are known to spontaneously and stably self-organize into native and non-native complexes [26], and they can then be cross-linked. Within 1 h of cross-linking, bands consistent with CenH3 tetramers and octamers are present in both experiments (Figure 2E, lanes 1–2, and Figure S1B, lanes 1–4), indicating that DMS can efficiently cross-link these species. Furthermore, the CenH3 antibody can detect its epitope reliably in cross-linked species above the size of a heterotypic tetramer, including an octameric species in vitro (Figure 2E, lane 2, band 8, and Table 1). The production of CenH3 octamers in vitro, using either native or recombinant CenH3, extends the results of Yoda and coworkers [16] for CENP-A and confirms that CenH3-containing octamers can be cross-linked under the conditions that failed to reveal their presence in unsynchronized cells.

The detection of tetramers and octamers in these in vitro cross-linking experiments (Figure 2E and S1B) contrasts with the detection of only CenH3-containing tetramers and dimers in vivo (Figure 2B, 2C, and S1A). Therefore, whereas CenH3 has the potential to form octamers and homotypic tetramers in the absence of DNA in vitro, it appears that in unsynchronized cells, the maximally cross-linked form of the CenH3 nucleosome is a tetramer, rather than a canonical octamer.

### CenH3 Interphase Tetramers Are Heterotypic and Contain H2A, H2B, and H4

**Figure 3:**
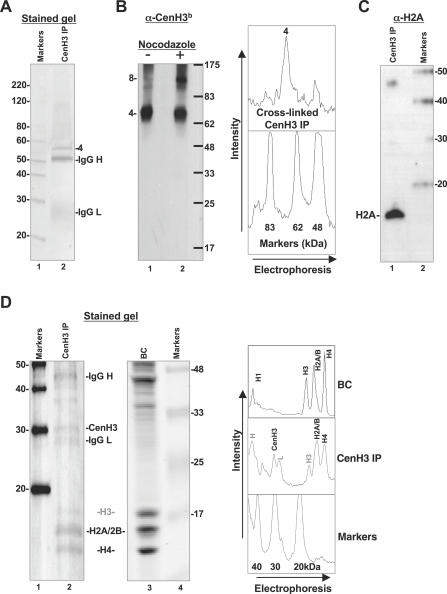
CenH3 IP Particles Are Primarily Stable Heterotypic Tetramers at Interphase Containing H2A, H2B, and H4 in Approximately Equimolar Amounts (A) A laser scan of SYPRO-Ruby stained 18% SDS-PAGE gel of cross-linked CenH3 IP particles shows a major species at 68 kDa. Similar formaldehyde cross-linked samples were analyzed by mass spectrometric analysis, and found to contain H2A, H2B and H4 (Table S1). IgG H and IgG L are heavy and light chain of the primary antibody used in the immunoprecipitation. Densitometric analysis to the right depicts an independent immunoprecipitation with similar result. Protein standard is Magic Marker, 20–220 kDa (Invitrogen, USA). (B) Western blot of cross-linked CenH3 IP particles probed with biotinylated anti-CenH3 antibody (α-CenH3^b^) detects heterotypic species 4 as the predominant species (lane 1). Nocodazole treatment of cells yields heterotypic species 4 as the dominant species, with the appearance of an octamer-size species (lane 2). Protein standard is pre-stained broad range marker 6.5–175 kDa (Invitrogen). (C) Detection of H2A in CenH3 IP particles. Formaldehyde cross-linked CenH3 IP particles (as in [B]) were boiled to reverse cross-links, and samples were electrophoresed on an 18% SDS-PAGE gel. The blot was probed with biotinylated anti-H2A antibody (Upstate catalog number 07–146). Protein standard is Magic Marker 20–220 kDa (Invitrogen). (D) CenH3 IP nucleosomes contain H2A, H2B, CenH3, and H4. A laser scan of a SYPRO-Ruby–stained 20% SDS-PAGE gel containing denatured CenH3 IP nucleosomes reveals approximately equimolar amounts of CenH3, H2A, H2B, and H4, and depletion of H3 (lane 2, and densitometric analysis to the far right), whereas the lane containing bulk chromatin (BC, lane 3, and densitometric analysis to the far right), resolved on the same gel, has normal core histone stoichiometry. Two protein standards were present on the gel: Magic Marker 20–220 kDa (Invitrogen) and pre-stained broad range Marker 6.5–175 kDa (Invitrogen).

The maximally cross-linked CenH3 nucleosomal species we observed in the cross-linking experiments in vivo (Figure 2B and S1A) migrated at approximately the correct molecular weight predicted for a heterotypic tetramer. However, potential anomalous migration of histone complexes relative to molecular weight markers could result in incorrect assignment of the species. We wanted to assess directly whether this species is heterotypic (i.e., CenH3, H2A, H2B, and H4) or homotypic (i.e., two copies each of CenH3 and H4). This experiment could not be done directly in chromatin extracts, because probing with antibodies to H2A or H2B would yield the entire range of bulk chromatin nucleosomal interactions, rather than specifically the CenH3 nucleosomal fraction. Therefore, we purified the limit cross-linked 68-kDa species by cross-linking overnight within the same amount of nuclei as before, using the reversible primary amine cross-linker formaldehyde (DMS cross-links are not reversible). We stripped cross-linked nucleosomal particles off genomic DNA with high-salt buffer, and CenH3 particles were immunoprecipitated. The CenH3 immunoprecipitated (IP) particles were eluted off the beads, concentrated by precipitation with trichloroacetic acid, and directly resuspended in SDS-PAGE loading buffer. Analysis of a portion of these cross-linked CenH3 species by staining with SYPRO Ruby and laser-based detection (Figure 3A, lanes 1–2) and by protein blots probed with a biotinylated antibody to CenH3 (α-CenH3^b^, Figure 3B, lane 1), confirmed that we could purify the dominant cross-linked 68-kDa CenH3 nucleosomal species observed in vivo.

As described above, we also attempted to obtain maximally condensed chromatin by incubating cells with nocodazole, a mitotic spindle–inhibiting drug. Cross-linking within these cells again yielded predominantly the approximately 68-kDa species (Figure 3B, lane 2), but also a minor amount of a second species at approximately 130 kDa, albeit in the absence of homotypic tetramers or hexamers. It is tempting to speculate that mitotic compaction led to the formation of a CenH3-containing octamer, or that kinetochore proteins associated with centromeric chromatin during mitosis cross-link with CenH3 to yield an approximately 130-kDa product.

To identify components of the 68-kDa tetramer cross-linked species above, we excised a gel slice containing this species and subjected it to in-gel trypsin digestion followed by mass spectrometric analysis (Figure 3). This approach positively identified peptides from H2A, H2B, and H4, but not H3, directly within the 68-kDa band (Protocol S1 and Table S1). We boiled the rest of the cross-linked sample to reverse formaldehyde cross-links and then probed for denatured H2A on a Western blot, confirming its presence (Figure 3C, lane 1).

Finally, we also resolved constituent histones within native CenH3 IP nucleosomes by denaturing SDS-PAGE, visualized by laser excitement of the SYPRO-Ruby stained gel (Figure 3D, lane 2, and densitometric scan to the far right). Roughly equimolar amounts of H2A, H2B, and H4 were present in the CenH3 IP fractions, whereas H3 was substantially depleted compared with the bulk chromatin fraction electrophoresed on the same gel (Figure 3D, lane 3), where all four core histones are present. The small amount of H3 present in the CenH3 IP lane likely originates from contaminating bulk nucleosomes present on the CenH3 IP beads.

Our mass spectrometric analysis identifying H2A, H2B, and H4 within the cross-linked CenH3 nucleosome (Protocol S1 and Table S1) and denaturing gel analysis of CenH3 IP particles demonstrating equimolar amounts of H2A, H2B, and H4 (Figure 3D) are consistent with past studies that have reported that H2A, H2B, and H4, but not H3, are associated with CenH3 in vivo [23,24]. From these analyses, we conclude that the limit cross-linked CenH3 nucleosomal species observed in vivo (Figure 2B, 2C, and S1A) is a heterotypic tetramer containing one copy of CenH3, H2A, H2B, and H4, rather than a homotypic tetramer containing two copies of CenH3 and H4.

### CenH3 Nucleosomes Have Reduced Nuclease-Protected DNA Wrapped about Their Surface

In bulk chromatin, DNA is wrapped in approximately two 80-bp turns around the symmetrical heterotypic halves of the canonical octameric nucleosome. Given the protein cross-linking data, which suggests that CenH3 nucleosomes are tetrameric, one would predict that there is less DNA wrapped about each CenH3 nucleosome compared with the two turns accommodated by a full octameric species. Previous studies have indicated that a reduced amount of DNA (approximately 120 bp) is wrapped about the CenH3 nucleosome in fission yeast [27] as well as in vitro [16]. Therefore, we wondered how much DNA is constrained by fly CenH3-nucleosomes. Traditional Southern blot analysis of nuclease-digested chromatin samples is unfeasible, because fly centromeres are thought to be composed of highly repetitive 5-bp satellite DNAs [28], unlike the unique sequences that make up budding and fission yeast centromeres. To circumvent this problem, we immunoprecipitated and eluted CenH3-containing oligonucleosomes from MNase-solubilized chromatin to assess directly its nuclease digestion pattern compared with that of either bulk or control IP chromatin.

**Figure 4:**
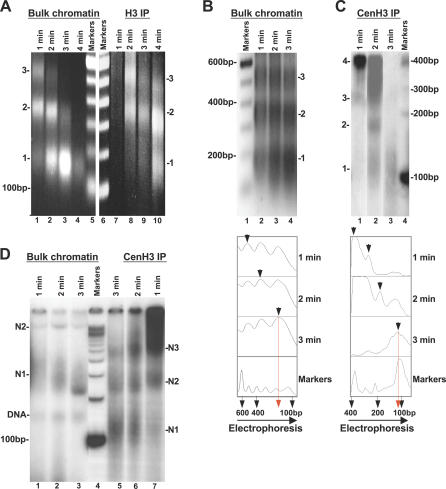
Less DNA Is Protected from Nuclease Digestion of CenH3- Compared with H3-Nucleosomes (A) Ethidium bromide stained 1.5% agarose gel showing DNA obtained from progressively MNase-digested bulk input chromatin (lanes 1–4) and control H3 IP chromatin (lanes 7–10). Both show core particle protection at 150 bp. Marker is 100-bp DNA ladder (Invitrogen). (B) Ethidium bromide stained gel showing purified DNA obtained from dilute MNase digestion of bulk chromatin input has a normal progression yielding a canonical MNase “ladder” and core protection of 150 bp. Marker is 100-bp DNA ladder (Invitrogen). The densitometric scan is presented below. (C) Autoradiogram showing purified DNA obtained from CenH3 immunoprecipitation of MNase-digested chromatin (lanes 1–3). DNA was radioactively end-labeled and resolved on 8% PAGE. The nucleosomal ladder appears relatively smeary and multiples of a unit repeat are not evident. Marker is 100-bp DNA ladder (Invitrogen). Densitometric analysis below demonstrates reduction in CenH3 core protection to approximately 100–120 bp rather than 150 bp seen for bulk chromatin (B). Lane 2 required a lower exposure than the rest of the gel. Marker is 100-bp DNA ladder. (D) Native PAGE of CenH3 IP nucleosomes obtained from successive time points of MNase-digested input (from [B], lanes 1–3) displays faster migration kinetics for CenH3 mononucleosomal species compared to BC species. Nucleosomes are radioactively end labeled in all lanes. Half of the gel containing CenH3 IP (lanes 5–7) needed a 5× longer exposure time than BC (lanes 1–3). The sharp band (DNA) is most likely free mononucleosomal DNA, and species N1, N2, and N3 refer to successive nucleosomal species. Marker is 100-bp DNA ladder.

We first assessed control H3 IP chromatin compared with bulk input chromatin. Across the time course of MNase digestion, comparing the input bulk chromatin to control H3 IP chromatin, the progression of the DNA ladder is similar (Figure 4A, bulk chromatin, lanes 1–4 versus H3 IP, lanes 7–10), with nuclease nicking linker DNA between regularly spaced nucleosomes creating a “ladder” of multiples of a unit repeat [29,30]. Moreover, protection of 150 bp [30] is seen in the H3 IP (Figure 4A, lane 10), suggesting that immunoprecipitation conditions do not detectably affect the chromatin at the level of nucleosomal DNA.

We next electrophoresed and purified CenH3 IP nucleosomal DNA from the same MNase-digested chromatin extracts to investigate nucleosomal spacing and core protection. However, under the conditions of digestion above (Figure 4A), the CenH3 pattern was over-digested and smeary, with no distinct bands apparent (unpublished data). To better resolve the kinetics of nuclease digestion of CenH3 chromatin, we used diluted MNase across the same time course (1, 2, 3 min), wherein BC samples progress from 185 × 3, 185 × 2, 185 × 1 down to the core protection of 150 bp (Figure 4B, lanes 2–4 and red arrow on the densitometric scan below). These inputs were used to immunoprecipitate CenH3 nucleosomes. The analysis of proteins within the CenH3 IP nucleosomes confirmed that they contain CenH3, H2A, H2B, and H4 (Figure 3D, lane 2), suggesting that the CenH3 IP nucleosomes were not disrupted.

We assessed purified DNA obtained from these CenH3 IP nucleosomes. Markedly, the CenH3 IP nucleosomal DNA pattern progresses directly from 185 bp to 120 bp, with no robust mono-nucleosomal core protection at 150 bp (Figure 4C, lane 3, and scan and red arrow marking monomer on densitometric scan below), suggesting that less DNA is afforded protection by wrapping about the CenH3 nucleosomal core. Furthermore, the CenH3 nucleosomal ladder does not appear to possess multiples of a unit repeat (generated by evenly spaced linker DNA) seen in bulk chromatin. The reduced core protection of approximately 120 bp and the smeary nucleosomal ladder are both reminiscent of that previously reported for centromeric chromatin in Chromosome 1 of fission yeast [27].

We also performed native PAGE of CenH3 IP nucleosomes (Figure 4D) to assess their progression from oligonucleosome to mononucleosome. Although the enrichment of CenH3 mononucleosomes at the expense of the higher species as the MNase time course progressed was similar to that in the input, there were differences in electrophoretic migration. The CenH3 nucleosomal species (Figure 4D, bands N1, N2, and N3, lanes 5–7) migrated ahead of the equivalent BC species (Figure 4D, bands N1 and N2, lanes 1–3), indicating that these particles may be smaller or more compact than the corresponding BC particle. The sharp band at 150 bp present on the BC portion of the gel (Figure 4D, band D, lanes 1–3) is most likely free mono-nucleosomal DNA, sometimes released on native gels. Faster migration kinetics of CenH3 mononucleosome would be consistent with a CenH3 nucleosome that has both less DNA (as determined from Figure 4C) and less protein (as deduced from the cross-linking experiments in Figure 2B–2D and Figure S1A) compared to canonical nucleosomes.

Overall, these data confirm that digestion kinetics of fly CenH3 chromatin are different from bulk chromatin, as has been reported previously for centromeric chromatin from other species [18,20,27]. Reduced core particle protection of 120 bp and faster migration kinetics of the CenH3 mononucleosome on native PAGE are consistent with a smaller core particle that is a tetrameric, rather than an octameric, species.

### CenH3 Nucleosomes Are Discrete “Beads on a String” and Resist Ionic Condensation

**Figure 5:**
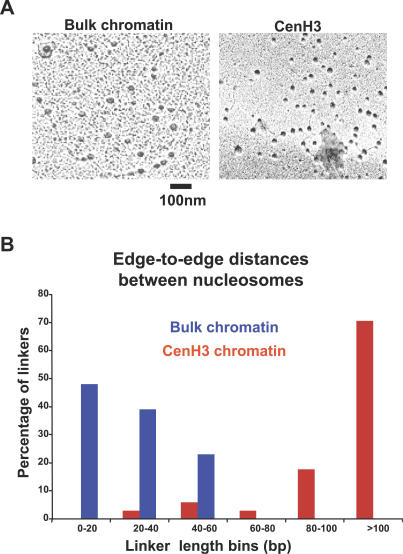
CenH3 Arrays Display Native Chromatin “Beads on a String” Appearance with Unusual Condensation Behavior (A) Nucleosomes of bulk chromatin are uniform-sized “beads” or particles with regular short linker DNA in low ionic strength buffer (left panel). Purified CenH3 IP nucleosomal arrays (>90%) display native chromatin's “beads on a string” appearance, but extended linker DNAs between CenH3 nucleosomes are suggestive of resistance to condensation under physiological salt conditions (right panel). The 100-nm bar was derived from magnification factors. (B) Edge-to-edge internucleosomal distances measured from two independent experiments using NIH Image J demonstrate that the more extended CenH3 linker DNAs are 2–3 times longer than bulk nucleosome linker lengths under comparable conditions.

We wondered if CenH3 nucleosomes are unstable octamers, perhaps falling apart into two stable halves, split open such that they have the biochemical characteristics of half-nucleosomes [31]. To test this possibility, we examined purified CenH3 chromatin by transmission electron microscopy. First, we assessed bulk chromatin, wherein we could recapitulate the characteristic appearance of native chromatin consisting of individual nucleosomes seen as distinct round particles along the DNA (“beads on a string”) [32,33] in low ionic strength buffer (Figure 5A, left) and characteristic chromatin structure with condensed arrays of uniform-sized nucleosomes under physiological ionic conditions (Figure S2A). The majority of rotary shadowed bulk nucleosomes (Figure S2B, 60%) were approximately 160 Å in diameter, within the range of expected nucleosomal diameter of 110 Å. A fraction (Figure S2B, bins >240 Å) contained larger particles representing clumped dinucleosomes or other aggregates.

Under physiological conditions, more than 90% of purified CenH3 chromatin arrays observed displayed distinct, well-separated, uniform-sized particles along the DNA consistent with native chromatin (Figure 5A, right), not disassembled structures, a result supported by the native PAGE results (Figure 4D). A small fraction (<10%) of CenH3 nucleosomal arrays contained aggregated structures (unpublished data) that were too large to be single nucleosomes and were excluded from the analysis. On a single grid, where variation should be minimal, particles in CenH3 IP arrays were found to fall primarily into two size classes (Figure S2B): a majority (>60%) fell into a class that consisted of 80-Å particles, with a minority ranging in size from 120 Å (20%) to 160 Å (<10%). In principle, the slightly smaller size class (80 Å) could represent CenH3 tetramers that we observed in all the cross-linking ladders, whereas a trace amount of contaminating H3 octameric nucleosomes on the same grid could account for the larger particles.

Finally, greater than 90% of CenH3 arrays appeared to be extended networks that are not condensed under physiological ionic conditions (Figure 5B and S2B). The linker DNA between CenH3 particles appears nonuniform in length, unlike canonical 20–40-bp linkers in this organism, which are highly regular (Figure 5A, left). Although it is not possible to directly assess compaction or higher-order structure by electron microscopy, the contour length of the more extended CenH3 linker DNAs was on average about 100 bp, or 2 to 3 times that of the more regular bulk linker DNA under comparable conditions (Figure 5B and S2B). The more extended CenH3 chromatin fiber relative to H3 chromatin is consistent with the observation that stretched fibers of alternating CenH3 and H3 arrays showed an apparently lower density of nucleosomes within the CenH3 chromatin [23]. These extended chromatin features might indicate higher order structural differences between CenH3 and bulk chromatin.

### Native CenH3 Nucleosomes Are Half the Height of Control Nucleosomes

It remained possible that the 80-Å CenH3-containing particles seen by electron microscopy reflect unusual shadowing effects upon these nucleosomes rather than the existence of a smaller particle. Therefore, we used atomic force microscopy (AFM) to measure the heights of CenH3-containing chromatin and control bulk chromatin that had been similarly obtained by affinity purification of biotin-tagged H4-containing nucleosomes. AFM is performed on soluble chromatin that attaches to a functionalized mica substrate and thus can provide direct measurements of particle dimensions [34]. Of particular interest to us are heights, because tetrameric nucleosomes are only half as high as octameric nucleosomes when they deposit on the mica substrate with protruding internucleosomal DNA [35].

**Figure 6:**
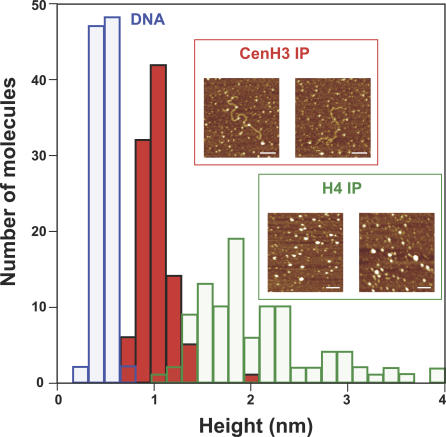
CenH3 Nucleosomes Are Half the Height of Bulk Nucleosomes as Measured by AFM Histogram depicting mean heights of control H4 IP nucleosomes (green bars) and CenH3 IP nucleosomes (red bars) computed from 100 counts of each. Mean H4 IP nucleosomal height is 2.05 ± 0.62 nm, in accordance with previously observed heights for bulk octameric nucleosomes. Mean CenH3 IP nucleosomal height is 1.03 ± 0.17 nm, or one-half octameric nucleosomal height, consistent with a tetrameric structure. Insets depict representative CenH3 IP molecules (inset, red box) and H4 IP molecules (inset, green box) used in this analysis. DNA heights (blue bars) were computed as an internal control for CenH3 IP nucleosomes. Scale bar represents 100 nm.

Height measurements were made of immunoprecipitated H4 and CenH3 nucleosomes and internucleosomal DNA fibers. The results are striking (Figure 6): whereas bulk nucleosomes display a range of heights that is consistent with expectation (mean 2.05 ± 0.62 nm, *n* = 100) [34], CenH3 nucleosomes are tightly distributed around a value that is half the mean of bulk nucleosomes (mean 1.03 ± 0.17 nm, *n* = 100). We conclude that CenH3-containing nucleosomes are predominantly tetramers in vivo.

## Discussion

We used protein cross-linking, compositional analyses, nuclease digestion, electron microscopy and AFM to characterize the structure of CenH3 nucleosomes in vivo. CenH3 nucleosomes display a noncanonical organization enriched in tetramers consisting of CenH3, H4, H2A, and H2B, which wrap no more than 120 bp of DNA. Importantly, AFM measurements of native CenH3 nucleosomes indicate that they are half the height of canonical nucleosomes, providing direct evidence for a tetrameric structure.

The tetrameric structure of interphase CenH3 nucleosomes is in sharp contrast to canonical nucleosomes, which are octameric, with no compelling evidence that they ever form stable heterotypic tetramers in vivo [31]. A problem with envisioning the existence of “half-nucleosomes” has been that this requires splitting octamers into halves, e.g.,, after replication to propagate epigenetic memory [36,37]. However, no processes are known that can separate the H3-H3 four-helix bundle interface to make half nucleosomes in vivo. In contrast to the replication-coupled assembly of bulk histones, CenH3 nucleosome assembly appears to occur predominantly at the G2/M phase of the cell cycle. CenH3-H4 dimers are assembled in chromatin by a single chaperone protein [17], whereas bulk chromatin is assembled by CAF-1 complexes, which evidently deposit two H3-H4 dimers in rapid succession to form a full nucleosome while tethered to proliferating cell nuclear antigen (PCNA) processivity factors at the replication fork [38]. Presumably, an untethered chaperone would deposit only single CenH3-H4 dimers. Therefore, we favor the possibility that CenH3 nucleosomes are formed by the deposition of a single CenH3-H4 dimer and a single H2A-H2B dimer, rather than being the product of splitting of pre-existing nucleosomes.

It is possible that the interphase CenH3 tetramer is also the form that organizes a kinetochore at mitosis. Alternatively, CenH3 tetramers might form higher-order complexes with other kinetochore proteins, or coalesce into octamers—either possibility is consistent with our detection of an approximately 140-kDa cross-linked complex in mitotic cells. The assembly of CenH3 octameric nucleosomes in vitro [16] and the conservation of residues that would form a CenH3 4-helix bundle [2] are consistent with condensation of tetramer pairs to form octamers for mitotic function. But in either case, our inability to detect a higher-order form in unsynchronized cells would require explanation. It is possible that only a small percentage of tetramers coalesce into higher-order structures at mitosis. The number of CenH3 nucleosomes required for kinetochore function might be very few: a single Saccharomyces cerevisiae Cse4-containing nucleosome is evidently sufficient to attach a single microtubule [1]. About 25 microtubules attach to typical mammalian kinetochores [39], and in *Drosophila* S2 cells, only about 11 microtubules are used to pull each chromosome to a pole [40]. Accordingly, the several thousands of CenH3 nucleosomes that we estimate to be present in a *Drosophila* kinetochore would be 2 to 3 orders of magnitude in excess of the number needed for it to attach to spindle microtubules at mitosis. Therefore, our inability to detect octamers in unsynchronized cells might simply reflect their low abundance relative to tetramers.

In addition to CenH3 that deposits at centromeres, several lines of evidence suggest that CenH3 can also be deposited on chromosome arms. During the pachytene stage of male meiosis in Arabidopsis thaliana, low levels of CenH3 are detected on chromosome arms [41]. In other cases, overexpressed CenH3 is deposited during interphase but is evicted before mitosis. CenH3s are assembled into nucleosomes by chaperones that are not specific for centromeres [17,42], and mislocalized CenH3s are removed by proteolytic degradation [6,7]. In *Drosophila,* CenH3 deposits promiscuously throughout chromosomes during G2, but then is progressively lost from chromosome arms while being retained at centromeric regions [6]. Efficient loss of CenH3 depends on the presence of proteasome components in both yeast and flies [6,7], which suggests that a continual process of CenH3 eviction operates to release the histone from chromatin for proteolytic degradation, and that this process evolved early in eukaryotic evolution. The tetrameric structure of interphase CenH3 nucleosomes should be energetically favorable for eviction relative to octamers. Eviction of canonical histones occurs at high levels throughout chromosome arms during interphase [43,44,45], and this process should evict tetrameric nucleosomes even more efficiently. Histone turnover is undetectable at pericentric heterochromatin [45], and this might allow for CenH3 accumulation. Indeed heterologous CenH3s accumulate in heterochromatin of *Drosophila* and human cells [25], and when gradually depleted for endogenous CENP-A, human centromeres can be maintained by yeast Cse4 as its only source of CenH3 [46]. Excess CenH3 deposited at pericentric regions might be destined for eviction by pulling forces during mitosis [4,47,48]. By this scenario, the presumptive higher-order form that we detect in cells arrested with nocodazole might be CenH3 that remains at kinetochores after eviction of CenH3 tetramers from chromosome arms.

We speculate that the quiescence of heterochromatin is an adaptation to prevent the eviction of CenH3 tetramers at the functional centromere. It has long been an enigma as to why centromeres are embedded in heterochromatin, even though they lack heterochromatic features themselves [47,49,50,51]. Thus, the need to allow eviction of CenH3 genome-wide, yet allow retention at kinetochores, provides a mechanistic rationale for the almost universal organization of centromeres within heterochromatin.

## Materials and Methods

### Isolation of input bulk chromatin.

D. melanogaster Kc167 or S2 cells (100 ml) were grown to mid-log phase [52]. For mitotic arrest, 10 μg/ml nocodazole was added to mid-log phase cells and incubated overnight alongside untreated cells. Cells were pelleted at low speed, rinsed twice with ice-cold 1× phosphate-buffered saline (PBS) buffer supplemented with 0.1% Tween, then suspended in 5–10 ml of ice-cold TM2 buffer (20mM Tris, pH 8.0, 2 mM MgCl_2_). NP-40 (100 μl) was gently mixed in to release nuclei, and cells were immediately put back on ice for an additional 2 min. Released nuclei were spun down at 800 revolutions per minutes (rpm) in an ice-cold Sorvall rotor for 10 min, rinsed once with fresh TM2 buffer [25], and re-suspended either in HB nuclear buffer [29] or in 0.5–1 ml 0.1M TE (10 mM Tris, 1mM EDTA, 100 mM NaCl) for subsequent MNase digestion. MNase (Sigma, http://www.sigmaaldrich.com) stock (2 units/5 μl) was diluted 10- or 25-fold in double distilled water. A sample of 5–10 μl of the diluted enzyme was added to nuclei prewarmed 5 min at 37 °C. Time points of digestion were 0.5, 1, 2, 3, 4, and 8 min at 37 °C in 2 mM CaCl_2_. Aliquots were removed and plunged in 5 mM EDTA on ice. MNase-digested nuclei were spun down at 1500 rpm, re-suspended in ice-cold nuclear extraction buffer (1× PBS supplemented to 0.35 M NaCl/2 mM EDTA/0.5 mM Phenylmethanesulfonylflouride, pH 8.8), and left in an end-over-end rotary shaker at 4 °C for 4–16 h. The supernatant (soluble chromatin extract) was used as input bulk chromatin for cross-linking, IP, and subsequent steps, and nuclear debris was spun out at 8000 rpm. The range of ionic strengths of buffers used in this study was 0.1 M NaCl (low), 0.15–0.35 M NaCl (moderate), and 0.5–2 M NaCl (high).

### Nucleosomal cross-linking in vivo and in vitro.

Freshly made 1 mg/ml DMS (Sigma, USA) in extraction buffer was added to approximately 0.1 mg/ml chromatin extract (adjusted to pH 8.5–8.8 or pH 9.0–9.3 slowly by dropwise addition of 0.2 N NaOH in the same buffer and equilibrated 15–30 min on ice) in extraction buffer, or in situ to nuclei (1 mg/ml chromatin) in HB nuclear buffer for 0–3 h at room temperature. For more extensively cross-linked samples, 0.05%–0.1% formaldehyde was added to nuclei for several hours. The reaction was stopped with 10% trichloroacetic acid, the chromatin extracts precipitated on ice, pelleted, rinsed once with acetone, dried, re-suspended and heated in 1× SDS-PAGE loading buffer (LB) supplemented with 0.1 M dithiothreiotol (DTT) to prevent di-sulfide associations. For in situ reactions, nuclei were boiled directly in 1× SDS-PAGE LB/DTT, or extracted with 2 M NaCl buffer (before and after cross-linking) for subsequent immunoprecipitation. For the Western blot analysis of CenH3 IP particles, biotinylated antibodies (EZ-link NHS biotinylation kit, Pierce, http://www.piercenet.com) were used to prevent fortuitous detection of residual heavy and light chains present in the immunoprecipitate.

DMS cross-linked samples were boiled and loaded on 4%–12% gradient NuPAGE gels in MES buffer (supplied by Invitrogen, http://www.invitrogen.com) (Figure 2) or 18% Tris-glycine (TG) SDS-PAGE gels in TG buffer (Figure 3), for resolution of complexes, run at 150 V until the dye was at the end. Gels were transferred to Optitran (http://www.whatman.com/) membranes overnight at 120 mA, per the manufacturer's recommendations. Western blots were blocked in 10% milk-supplemented PBS with 0.1% Tween (MPBST) for 2 h, rinsed with PBS, incubated overnight in primary anti-Cid antibody at 1:1000 dilution in 2% MPBST, or anti-H3 (N-terminal 1–20, Upstate/Millipore, http://www.upstate.com) at 1:5000 dilution in 5% MPBST, rinsed, and incubated with secondary antibody (IRD-680 labeled anti-rabbit IgG or HRP-conjugated anti-rabbit IgG at 1:10,000 dilution for 2 h). Blots were rinsed three times with PBS, and either directly analyzed at 700 nm in a Li-Cor Odyssey scanner, or incubated 10 min in chemiluminescence substrate (Pierce, USA) and exposed to BioMax MS film (Kodak, http://www.kodak.com) for 0.5–2.5 min. Blots were stripped by shaking overnight at 65 °C in 0.2 M glycine, 0.1% Tween, and rinsed three times with PBS before re-use. Biotinylation of the primary antibody for Western analysis used streptavidin-HRP as the secondary antibody, thus avoiding promiscuous detection of residual heavy and light chain immunoglobulins from the IP that could interfere with analysis of histone-complexes.

Molecular weights of complexes were estimated by NIH Image J, Odyssey Imaging System (Li-Cor, http://www.licor.com) or Typhoon 9410 (Molecular Dynamics, http://www.ump.com/mdynamic.html) densitometric scans of blots, using the known molecular weights of the Magic Marker (20–220 kDa, Invitrogen, detected by secondary antibody on the blot), or pre-stained protein marker (6.5–175 kDa, Invitrogen, by carefully realigning the negative with the blot).

CenH3 particles were reconstituted in vitro by adding soluble His-tagged recombinant CenH3 to HPLC-purified H2A, H2B, and H4 at a 1 mg/ml equimolar mixture in 0.1% triflouroacetic acid, or directly using 2 M salt-extracted native histones from Kc cell nuclei. The salt concentration was stepped up to 0.1 M NaCl, 0.6 M NaCl, and 2 M NaCl (in 1× PBS) in the case of recombinant mixtures. Samples were incubated for 1 h on ice at each step. Samples were diluted to 0.1 mg/ml in high salt before cross-linking as above.

### Centromeric chromatin and H4-containing chromatin IP.

Soluble CenH3-nucleosomes were precipitated from nuclease-digested chromatin extracted with 1× PBS supplemented with 0.35 M NaCl/2 mM EDTA/0.5 mM PMSF (adapted from [53]), with peptide antibody raised to the fly CenH3 N-terminal tail at a dilution of 1:1000, for 12 h at 4 °C. The immune complexes were pulled down with hydrated Protein G Sepharose beads for 2 h (10% v/v). The IP was spun down gently (1500 rpm, or on sucrose cushions) and washed in ice-cold 1× PBS supplemented with 0.35–0.15 M NaCl/2 mM EDTA/0.5 mM PMSF three times for 15 min each in an end-over-end rotator. Biotin-tagged H4 nucleosomes [54] were immunoprecipitated with monomeric avidin (Pierce Biochemicals) in parallel with the CenH3 IP under identical conditions and washes, and eluted off avidin beads with 2 mM biotin-supplemented buffer. CenH3 particles and short CenH3-nucleosomal arrays were either boiled directly for protein analysis or eluted off the beads by mixing in 0.1 mg/ml CenH3-specific peptide for 2–4 h in an end-over-end rotator. The beads were then spun out at 8000 rpm, soluble chromatin collected and used directly in subsequent biochemical experiments. CenH3 chromatin was found to be stable in buffer containing at least 0.15 M NaCl to avoid disassembly, and used within 1 wk of storage.

A portion of the CenH3 IP, H3 IP, and BC nucleosomes were boiled in 1× SDS-PAGE loading buffer containing 5% β-mercaptoethanol or 0.1 M DTT, resolved on 18%–20% SDS-PAGE gels (Figure 3D), and stained with SYPRO-Ruby (Molecular Probes) for protein content analysis by scanning in at 488 nm or 600 nm in the Typhoon Phosphorimager.

A portion of the IP nucleosomes was radioactively end-labeled with T4 Polynucleotide Kinase (NE Biolabs, http://www.neb.com) and 50μCi γ^32^P-ATP for 30 min at 37 °C, and nucleosomal integrity assessed by electrophoresis on 4.5% native PAGE 5 cm × 7 cm gels in low ionic strength 0.25× Tris-borate-EDTA (TBE) buffer at 2 mAmp for 3 h (pre-run was 30 min) (Figure 4D). Gels were dried and auto-radiographed or scanned in the Typhoon Phosphorimager.

A portion of the IP nucleosomes was de-proteinized and the purified DNA radioactively end-labeled as above and resolved on 6%–8% PAGE/1× TBE gels (with a 30 min pre-run) to reveal MNase protection for CenH3 IP versus control IP (Figure 4A–4C). Gels were dried and exposed for 4–24 h with intensifier screens to Biomax MS (Kodak) film.

### Transmission electron microscopy and image analysis.

A JEOL 1010 transmission electron microscope (FHCRC Shared Resources, http://www.fhcrc.org/) was used at 80,000 eV. Purified CenH3 IP and BC samples were deposited on ultra-thin carbon-coated/Formvar/200 mesh copper grids (Electron Microscopy Sciences, http://www.emsdiasum.com) and subsequently rotary shadowed lightly with platinum-palladium alloy (Electron Microscopy Sciences). Longer CenH3-nucleosomal arrays were dialyzed against 0.15 M NaCl and then visualized by electron microscopy still attached to beads because of technical difficulties in eluting them successfully. Nucleosomal arrays were viewed at 40,000× (Figure 5A left), 60,000× (Figure 5A right) or 80,000× (Figure S2A) magnification and images recorded on electron microscopy film (Kodak) or on a GATAN 2 k × 2 k camera. Negatives were scanned in at 800-1200 dpi and images adjusted for brightness and contrast. Approximately 100 arrays were observed over three independent experiments. NIH Image J program was used to automatically count sizes of CenH3 IP and BC nucleosomal particles using “threshold”-adjusted images. The scale was set to angstroms based on the magnification bar for each image. The “analyze” function was set to count particles in the 10–350-Å range. The counts were exported to Microsoft Excel, and histograms computed based on 40-Å bins going from 80 Å to 320 Å. Edge-to-edge internucleosomal distances were computed by highlighting linkers and using NIH Image J “measure” function. The counts were exported to Microsoft Excel, and histograms computed based on 20-bp bins going from 0–120 bp.

### AFM measurements.

AFM measurements were performed on CenH3 IP and H4 IP particles in parallel. IP samples were deposited onto 2 μM glutaraldehyde-treated APTES-mica for 30 min as described [55]. In situ imaging was carried out with a Macmode PicoSPM (Agilent Technologies, http://www.agilent.com) equipped with Type II MAClevers (Agilent Technologies) with a spring constant of 2.8 N/m. The scanning rate was 1.78 Hz. 100 particles for each immunoprecipitate were counted. DNA height was computed as an internal control for CenH3 IP samples. Note that heights measured by AFM differ from crystallographic values because of sample compression and uncertainty about the baseline owing to adsorbed salts. AFM experiments and measurements were performed (by HW and SL) without prior knowledge of the biochemical findings described in this study.

## Supporting Information

Figure S1 Brief Cross-linking Detects Intranucleosomal Contacts in CenH3 Nucleosomes Western blots of lower time points (0–60 min) for cross-linking at lower pH (8.5–8.8) in chromatin extracts (A) and in vitro reconstituted particles using recombinant CenH3 (B). Anti-H3 (left) detects monomers, dimers, trimers, tetramers, and hexamers in less than 1 h of cross-linking, whereas duplicated samples probed with anti-CenH3 (right) detect only dimer and tetramer size products over the same time course. (B) Cross-linking of in vitro reconstituted recombinant CenH3 particles detected with the antibody to CenH3 display a full range of products over the same time course. Cartoons on left and right depict deduced species.
(1.8 MB PDF)

Figure S2 Centromeric Chromatin Is Extended Under Physiological Conditions Higher magnification views of bulk nucleosomes (A) condensed in moderate salt, and CenH3 IP nucleosomes extended under similar ionic concentrations. (B) Nucleosomal widths of rotary shadowed particles. NIH-Image J histograms calculating particle sizes from Figure 5A and 5B reveals uniform sized particles for both BC and CenH3 samples, with the majority of sample diameters within the nucleosomal range 80–160 Å.
(1.6 MB PDF)

Table S1 Mass Spectrometric Analysis of CenH3 Chromatin Detects H2A, H2B and H4 Peptides (112 KB PDF)

Protocol S1 Supplementary Materials (65 KB PDF)

## Abbreviations

AFM: atomic force microscopy
BC: bulk chromatin
CenH3: centromere-specific histone H3
DMS: dimethyl suberimidate
IP: immunoprecipitated
MNase: micrococcal nuclease
