# Supplementary material for: Tetrameric Structure of Centromeric Nucleosomes in Interphase Drosophila Cells
Source: PLoS Biol. 2007 Jul 31;5(8):e218. doi: 10.1371/journal.pbio.0050218 (PMC1933458; doi:10.1371/journal.pbio.0050218)
Supplement: Supplementary file 1 [file pbio.0050218.sd001.pdf]

Supplementary materials

Mass Spectrometry Analysis of CenH3 IP particles

Purified CenH3 IP material was electrophoretically separated on a 18-20% SDS-PAGE gel, individual proteins were either visualized by Coomassie Brilliant Blue stain excised and subjected to an in-gel tryptic digest (digests were performed as described (Shevchenko et al, 1996), or the eluted CenH3 particles were precipitated with 10% trichloroacetic acid, washed once with acetone, dried, re-dissolved in de-ionized water, and digested in solution with Arg-C protease. For CenH3 identification, we could not detect peptides from the protein directly in the gel slice containing the tetramer (confirmed by Western blot analysis). Therefore, we used eluted CenH3 protein from extensively washed CenH3IP-beads.

Following digestion, samples were desalted using a microC18 ZipTip (Millipore) and dried. Samples were then resuspended, in 5 μL of 0.1% formic acid and analyzed by LC/ESI MS/MS (Liquid Chromatography coupled to Electrospray Ionization tandem mass spectrometry) with a nano2D LC (Eksigent) coupled to an LTQ-FT mass spectrometer (ThermoElectron) using an instrument configuration as described (Yi et al, 2003). Data were collected in a data-dependent mode in which a high mass resolution/high mass accuracy scan (in the FT part of the instrument) was followed by MS/MS scans of the five most abundant ions from the preceding MS scan (in the LTQ part of the instrument). The five selected ions for tandem MS were placed on an exclusion list and not selected for subsequent tandem mass spectrometry for 1.5 minutes, allowing less intense ions to be interrogated for tandem mass spectrometry.

Proteins were identified from mass spectrometry data using a modified version of the open source X!Tandem (Beavis Informatics) automated protein database search algorithm. The score function of native X!Tandem was replaced with a dot-product based score algorithm that is compatible with Peptide Prophet (Keller et al, 2005). Search results were analyzed by the Peptide Prophet program and identifications were

made against an in silico digested library generated from proteins obtained from the fly database (FlyBase). Identifications were considered correct if at least two peptides had raw scores greater than 200 for +1 ions, 200 for +2 ions, and 300 for +3 ions, %Ions of greater than 15% and if the identification did not appear in a blank portion of the gel. Single peptide identifications were considered correct if they passed the above criteria and if they had Peptide Prophet scores greater than 0.90 (10% error rate). Under these criteria, histones H2a, 2b and H4, but not H3 were positively identified, with at least 2 unique non-overlapping peptides each, within the cross-linked CenH3 IP gel slice. The control (BC) gel slice positively identified all four core histone peptides. However, for CenH3 identification, the criteria had to be reduced to a less stringent Peptide Prophet score of greater than 0.5 (50% error rate), resulting in the identification of three unique large non-overlapping CenH3 peptides (see table) that we feel reasonably confident of its identity.

Supplementary References

1. Shevchenko, A., M. Wilm, O. Vorm, and M. Mann, Mass spectrometric sequencing of proteins silver-stained polyacrylamide gels. Anal Chem, 1996. 68(5): p. 850-8.

2. Yi, E.C., H. Lee, R. Aebersold, and D.R. Goodlett, A microcapillary trap cartridge-microcapillary high-performance liquid chromatography electrospray ionization emitter device capable of peptide tandem mass spectrometry at the attomole level on an ion trap mass spectrometer with automated routine operation. Rapid Commun Mass Spectrom., 2003. 17(18): p. 2093-8.

3. Keller, A., J. Eng, X. Zhang, and R. Aebersold, A uniform proteomics MS/MS analysis platform utilizing open XML file formats. Molecular Systems Biology, msb4100024, 2005.
