# Supplementary material for: Tetrameric Structure of Centromeric Nucleosomes in Interphase Drosophila Cells
Source: PLoS Biol. 2007 Jul 31;5(8):e218. doi: 10.1371/journal.pbio.0050218 (PMC1933458; doi:10.1371/journal.pbio.0050218)
Supplement: Supplementary file 2 [file pbio.0050218.sg001.pdf]

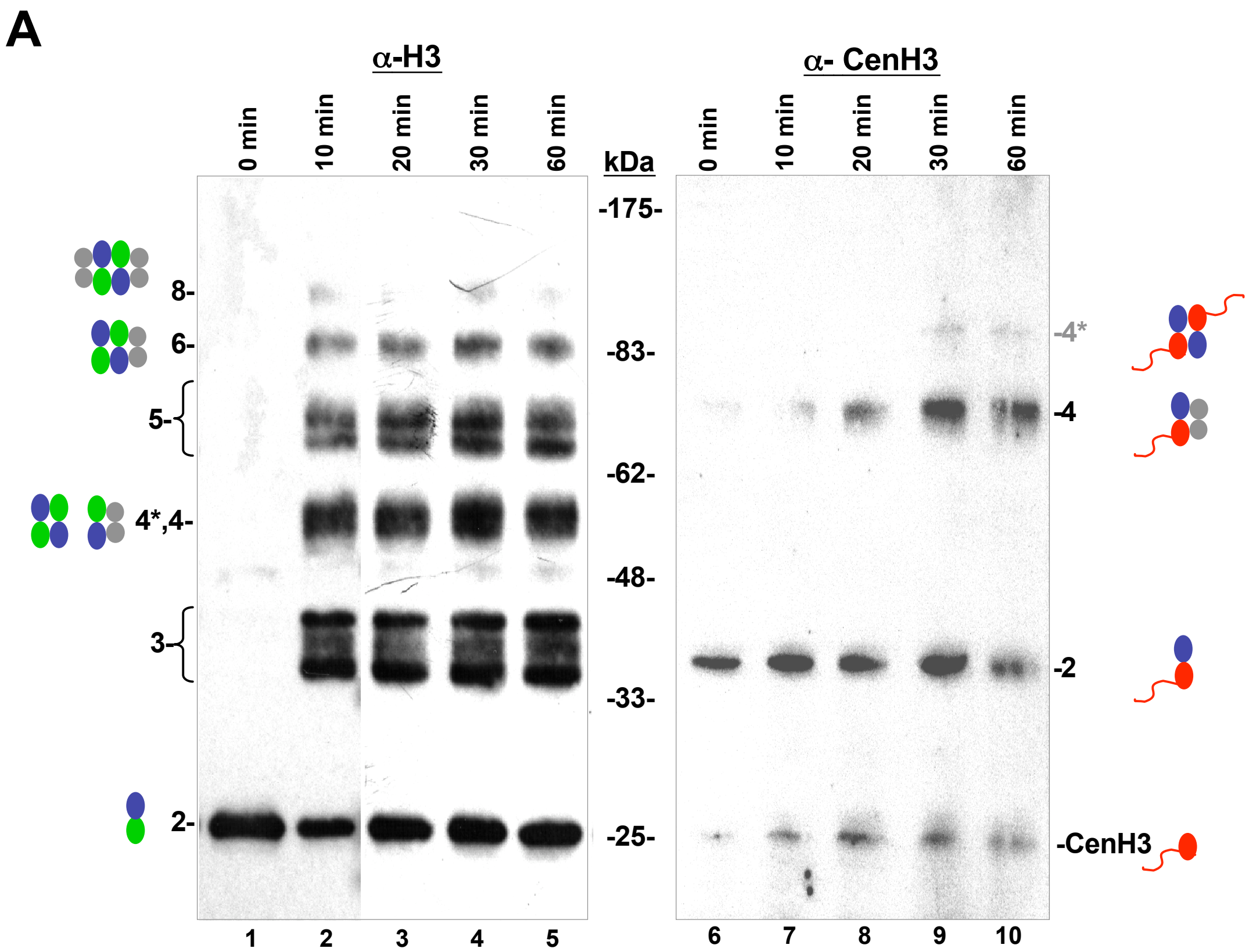

A
α-H3
0 min
10 min
20 min
30 min
60 min
kDa
-175-
8-
6-
-83-
5-
-62-
4*,4-
-48-
3-
-33-
2-
-25-
1
2
3
4
5
α- CenH3
0 min
10 min
20 min
30 min
60 min
-4*
-4
-2
-CenH3
6
7
8
9
10

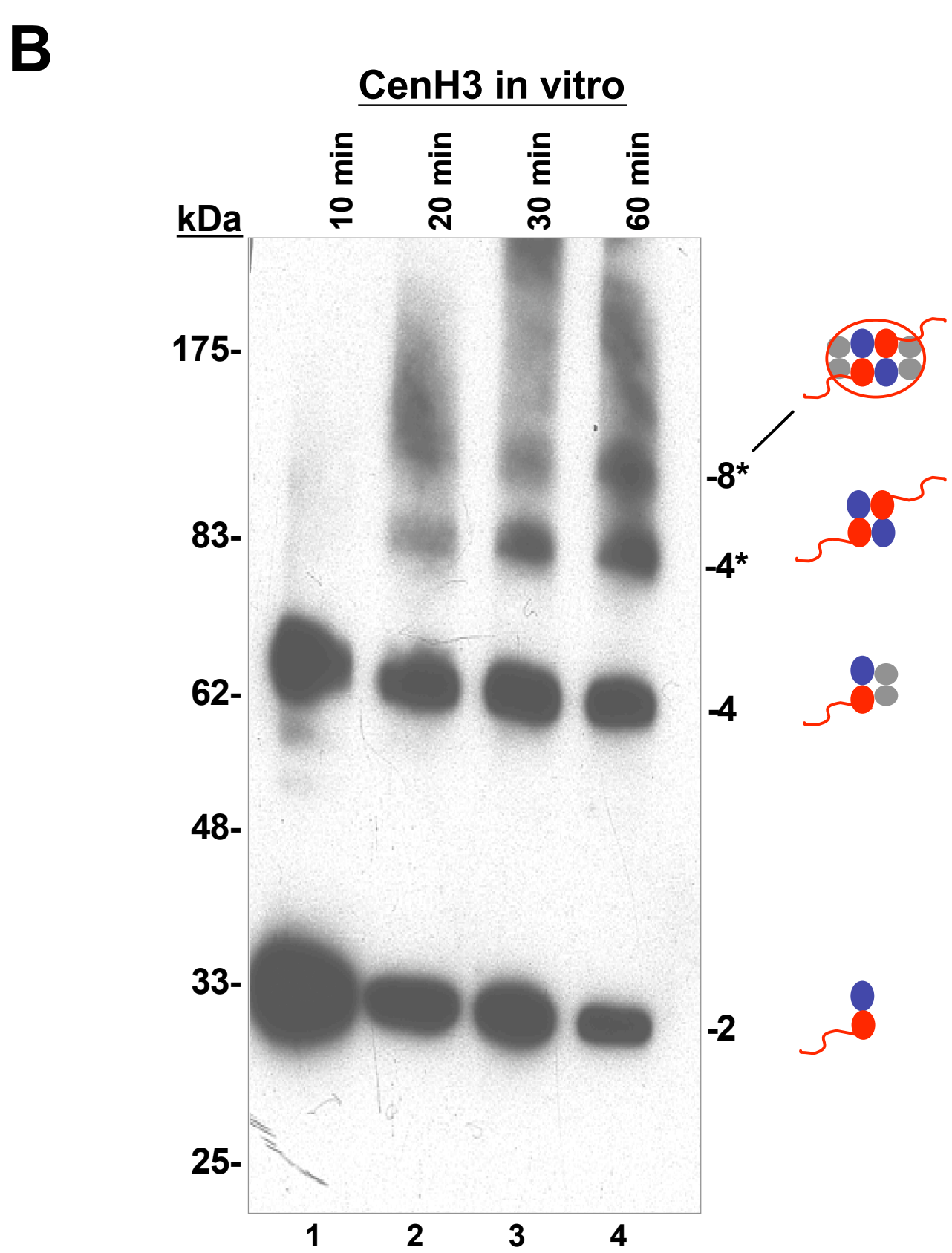

B
CenH3 in vitro
10 min
20 min
30 min
60 min
kDa
175-
83-
62-
48-
33-
25-
-8*
-4*
-4
-2
1
2
3
4

Figure S1
