# Supplementary material for: Tetrameric Structure of Centromeric Nucleosomes in Interphase Drosophila Cells
Source: PLoS Biol. 2007 Jul 31;5(8):e218. doi: 10.1371/journal.pbio.0050218 (PMC1933458; doi:10.1371/journal.pbio.0050218)
Supplement: Supplementary file 3 [file pbio.0050218.sg002.pdf]

A

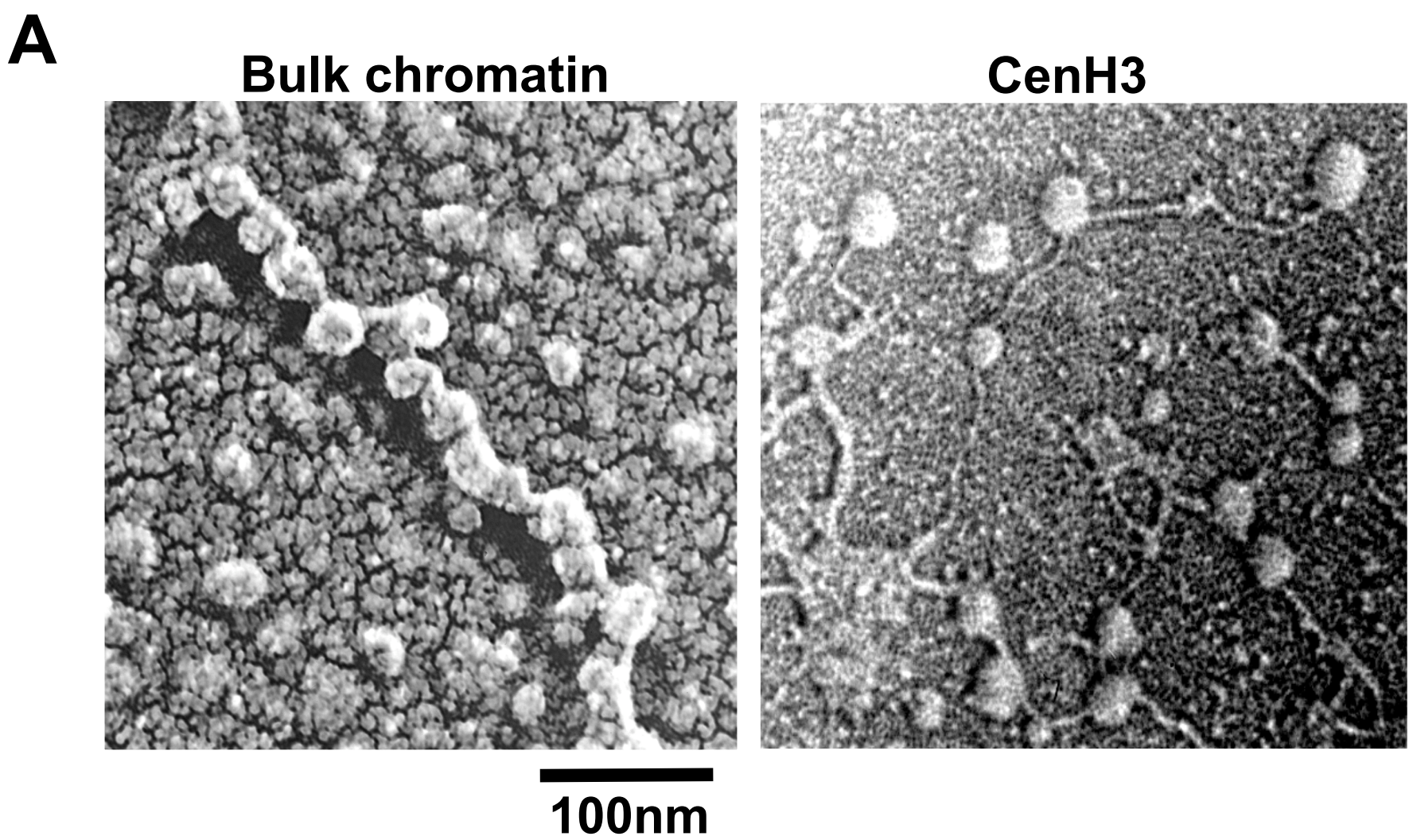
Bulk chromatin
CenH3
100nm

B

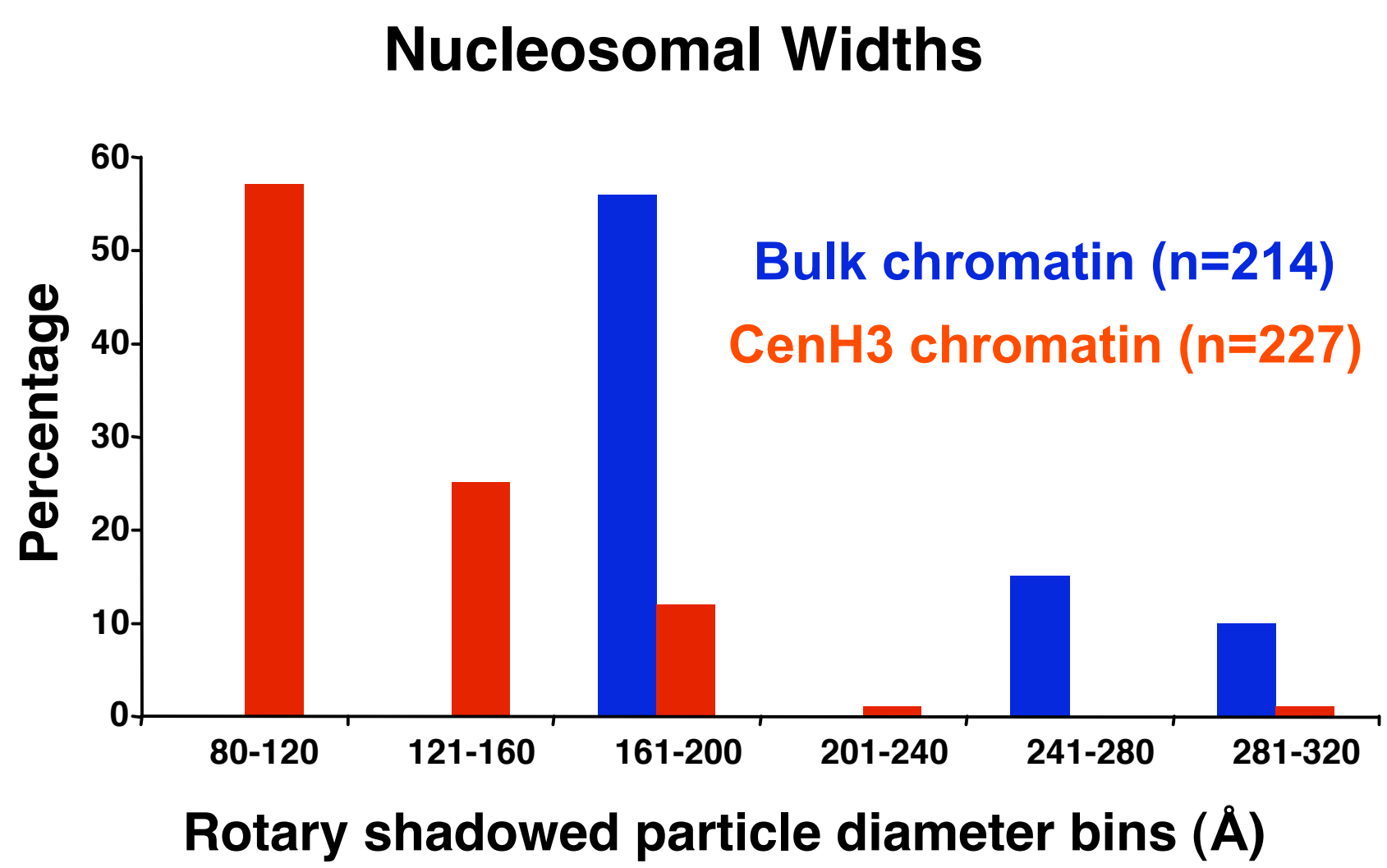
Nucleosomal Widths
Percentage
60
50
40
30
20
10
0
Bulk chromatin (n=214)
CenH3 chromatin (n=227)
80-120
121-160
161-200
201-240
241-280
281-320
Rotary shadowed particle diameter bins (Å)

Figure S2
