# Supplementary material for: Tetrameric Structure of Centromeric Nucleosomes in Interphase Drosophila Cells
Source: PLoS Biol. 2007 Jul 31;5(8):e218. doi: 10.1371/journal.pbio.0050218 (PMC1933458; doi:10.1371/journal.pbio.0050218)
Supplement: Supplementary file 4 [file pbio.0050218.st001.pdf]

Supplementary Table 1

LC/MS-MS and identification of proteins in CenH3 immune-precipitate

Experiment	Histone Proteins Identified	Peptides	Protein Prophet Score
CenH3 cross-linked tetramer Gel slice (Figure 3a)	Histone H2a	R.AGLQFPVGR.I	0.998
	Histone H2a	K.LLSGVTIAQGGVLPNIQAVLLPK	0.988
	Histone H2b	R.KESYAIYIYK.V	0.944
	Histone H2b	K.AMSIMNSFVNDIFER.I	0.998
	Histone H4	R.DNIQGITKPAIR.R	0.988
	Histone H4	R.ISGLIYEETR.G	0.997
CenH3 eluted from 2M NaCl-washed CenH3 IP beads	Cid	R.APRPSANNSKSPNDDDTAFR.S	0.545
	Cid	R.LQHHPGTLIPK.L	0.793
	Cid	R.LADSYM’LTK.H	0.502
*Control H3+2a+2b+ H4 Gel slice	Histone H2a	K.LLSGVTIAQGGVLPNIQAVLLPK.K	0.999
	Histone H2a	R.NDEELNKLLSGVTIAQGGVLPNIQA	0.999
	Histone H2b	VLLPK.K	0.999
	Histone H3	K.AM'SIM'NSFVNDIFER.I	0.995
	Histone H4	R.E^IAQDFKTDLR.F	0.999
	Histone H4	K.RISGLIYEETR.G	0.999
		R.KTVTAMDVVYALKR.Q	

*Mass spectrometric analysis of control H3/2a/2b/H4 bulk histone gel slice also identified several non-histone proteins which have not been shown.
